# The impact of ultrasound scan training on its usage among emergency personnel in Malaysian emergency departments

**DOI:** 10.1186/2036-7902-6-S1-A24

**Published:** 2014-01-31

**Authors:** JM Noor, FM Salleh

**Affiliations:** 1 Faculty of Medicine, Universiti Teknologi Mara, Malaysia; 2 Emergency Department, Hospital Sg Buloh, Malaysia

## Background

The use of ultrasound scan (USS) in Malaysian Emergency Departments (ED) began ten years ago but has increased exponentially over the last four years. The aim of our study is to determine the current training and usage of emergency ultrasound.

## Methods

A questionnaire was distributed to 47 hospitals with Emergency Physicians (EP) in Malaysia. Questions included patient load, background on USS courses attended and frequency of ultrasound usage in clinical management.

## Results

29 hospitals responded including 15 general hospitals, 11 district hospitals and 3 university hospitals. All ED has at least 1 ultrasound machine.

In general hospitals, patient load ranged between 60,000 – 320,000 per year. Mean number of EPs is 4. Mean percentage of EPs who had attended any course is 79% (SD 27.8). In 7 hospitals, 100% had attended a course. In 1 hospital, the sole EP has not attended an USS course. The average percentage of Medical Officers (MO) who had attended an USS course is 41% (SD 17.7). In terms of frequency of USS usage, 80% cited usage of ‘very often’ and 20% cited ‘often’.

In district hospitals, patient load varied between 45,000 -130,000 per year. Most EDs had at least 1 EP. Except for one EP, all had attended an USS course. 35% of MOs had attended an USS course (SD 32.9). The frequency of use cited was 30% as ‘very often’ and 70% as ‘often’.

Whilst in university hospitals, patient load was between 65,000 – 100,000 patients per year. The mean number of EPs was 11. An average of 53% of EPs had attended an USS course, but only 26% of MOs had attended a similar course. In terms of frequency of use, 67% cited ‘very often’ and 33% cited ‘often’.

All agreed that USS changed their clinical management significantly.

## Conclusion

Across the different hospital settings in Malaysia, all cited that USS is used ‘often’ and ‘very often’. However, there are more EPs and MOs in general hospital that has been trained in USS compared to university setting. This is in contrast with the study in California (2009) where most ED do not use ultrasound and academic EDs used it more than community EDs. The issue now is the credentialing process is not fully determined.

